# COVID‐19 sniffer dog experimental training: Which protocol and which implications for reliable sidentification?

**DOI:** 10.1002/jmv.27147

**Published:** 2021-06-26

**Authors:** Silvia Angeletti, Francesco Travaglino, Silvia Spoto, Maria Chiara Pascarella, Giorgia Mansi, Marina De Cesaris, Silvia Sartea, Marta Giovanetti, Marta Fogolari, Davide Plescia, Massimiliano Macera, Raffaele Antonelli Incalzi, Massimo Ciccozzi

**Affiliations:** ^1^ Unit of Clinical Laboratory Science University Campus Bio‐Medico of Rome Rome Italy; ^2^ Department of Emergency University Campus Bio‐Medico of Rome Rome Italy; ^3^ Department of Diagnostic and Therapeutic Medicine University Campus Bio‐Medico of Rome Rome Italy; ^4^ Head of the Drive In Area University Campus Bio‐Medico of Rome Rome Italy; ^5^ Laboratório de Flavivírus Instituto Oswaldo Cruz, Fundação Oswaldo Cruz Rio de Janeiro Brazil; ^6^ K9 Unit SecurityDogs NGS Private Security Company Rome Italy; ^7^ Gerontology Unit Campus Bio Medico University and Teaching Hospital Rome Italy; ^8^ Unit of Medical Statistics and Molecular Epidemiology University Campus Bio‐Medico of Rome Rome Italy

**Keywords:** COVID‐19, dog training, sniffer dog, VOCs

## Abstract

The introduction of trained sniffer dogs for COVID‐19 detection could be an opportunity, as previously described for other diseases. Dogs could be trained to detect volatile organic compounds (VOCs), the whiff of COVID‐19. Dogs involved in the study were three, one male and two females from different breeds, Black German Shepherd, German Shepherd, and Dutch Shepherd. The training was performed using sweat samples from SARS‐CoV2 positive patients and from SARS‐Cov2 free patients admitted at the University Hospital Campus Bio‐medico of Rome. Gauze with sweat was collected in a glass jar with a metal top and put in metal boxes used for dog training. The dog training protocol was performed in two phases: the olfactory conditioning and the olfactory discrimination research. The training planning was focused on the switch moment for the sniffer dog, the moment when the dog was able to identify VOCs specific for COVID‐19. At this time, the dog was able to identify VOCs specific for COVID‐19 with significant reliability, in terms of the number of correct versus incorrect (*p* < 0.0001) reporting. In conclusion, this protocol could provide a useful tool for sniffer dogs' training and their introduction in a mass screening context. It could be cheaper and faster than a conventional testing method.

## 1 INTRODUCTION

The dog olfactory system with the olfactory mucosa of the nasal cavity has been largely studied for its unique characteristic consisting of the presence of basal cells providing the regular regeneration of olfactory sensory neurons^1^ and for its abundance of olfactory receptor reaching about 200 million.^2^, ^3^ The dog's olfactory properties have been largely employed for the research and detection of explosive substances or dead bodies, as dog support units in police, army and civil protection divisions, in harbors and airport, and in the private security agency.

The use of sniffer dogs in medical settings can be dated back to 1989 and since then many other applications have been described, such as breast and lung cancers with a percentage of detection rate ranging from 88% to 99%^4^, a malarial disease with a detection rate of about 82%^5^, and viral or bacterial infections with a detection rate of 77%–92.6%.^6^, ^7^, ^8^

Several volatile organic compounds (VOCs) account for the odor released by the expiration phase of breathing, skin emanation, urine and breath vapors, saliva, and pathological conditions. These odors depend on biochemical modification occurring in the body with the consequent release of these specific compounds that are volatile.^9^ The metabolic changes occurring in the body in presence of specific conditions, such as inflammation, infections, or neoplastic disease can be recognized by the dogs that are provided by a powerful olfactory apparatus if adequately trained for detection of the VOCs.^10^

The same approach could be used for COVID‐19 detection, as described in previous studies.^11^, ^12^, ^13^, ^14^ The VOCs could be useful in clinical diagnosis of different disease including bacterial and viral infections as SARS‐CoV‐2 causing COVID‐19interstizial bilateral pneumonia.^11^, ^12^, ^13^, ^14^ In a recent study, dogs professionally trained were evaluated for glucose level detection in patients with diabetes. This study suggested that dogs, after adequate training, have the ability to detect hypo and hyperglycemic conditions.^15^

VOCs' detection‐trained dogs could provide early detection of SARS Cov‐2 infected patients at low cost. The trained dog has the ability to screen more than 200 individuals per hour, enough to allow mass screening at airports, stadiums, or in case of crowded events where the virus transmission control by asymptomatic individuals is fundamental. This is in agreement with World Health Organization recommendation about mass screening and its application also in low‐income countries where the use of sophisticated and expensive screening tools could be limiting.

The study aims to evaluate the sniffer dogs' ability to discriminate VOCs emanated by the skin in course of COVID‐19, demonstrating that this disease is characterized by a specific odor and that dogs are able to identify it efficiently and quickly.

## 2 MATERIALS AND METHODS

### 2.1 Experimental design

The training planning was developed involving dogs from different breeds. Dogs involved in the study were from different working dog breeds since their features are useful to standardize the characteristics, the management and the training coherence, to the advantage of more homogeneity in results recording. The intention was to concentrate the experiment on high quality rather than on the number of dogs. In fact, dogs were selected for their specific talents suitable to this kind of experimental design, such as temperament, docility, and resistance.

COVID‐19 conditioned dogs, once involved in the study, will be recoverted to other activities of safety and security, if necessary or at the end of the pandemic, to guarantee the service continuity and mental and physical dogs health in the future. The study was approved by the Local Ethics Committee of University campus Bio‐Medico of Rome (PAR 17.21 OSS).

The dogs' training plan was divided into two steps: the first step was “specific conditioning” to COVID‐19 VOCs, consisting of the association of the odor research and consequent reporting. This critical and fundamental step is developed using several sweat samples from patients admitted to the COVID Center of the University Hospital Campus Bio‐Medico of Rome, for COVID‐19. The second step of “olfactory discrimination research” consisted of the discrimination between the COVID‐19 odor of interest and everything else that has to be discarded. Here, the discrimination was performed between a box containing underarm sweat collected on gauze from SARS‐CoV2 positive patients, a box containing underarm sweat collected on gauze from SARS‐CoV2 negative patients, a box containing blank gauze, and an empty box. The different boxes were randomly positioned in a line‐up from a minimum of four possibilities upwards.

The training involved the repetition of different experimental sessions in the line‐up to fix more and more the VOCs in the olfactory memory of the dogs.

### 2.2 Sniffer dogs' characteristics

The dogs involved in this study belonged to the SecurityDogs, a brand of NGS srl Security company (Italy), one male and two females from three different breeds: Black German Shepherd, German Shepherd, and Dutch Shepherd. The demographic characteristics of dogs are reported in Table 1.

**Table 1:** Demographic characteristics of dogs involved in the study protocol

Dog's name	Age (year)	Gender	Specie	Explosive substances trained
Harlock	3	Male	Black German Shepherd	Yes
Roma	4	Female	Dutch Shepherd	Yes
Idra	1	Female	German Shepherd	No

### 2.3 Materials used for sweat samples collection and training

**Figure 1:**
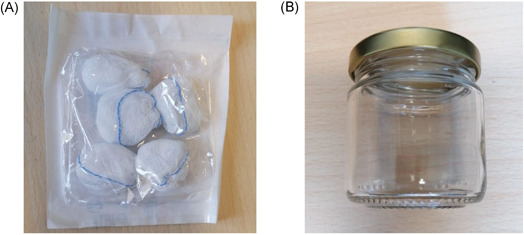
(A) Gauze used for underarm sweat collection by patients. (B) Glass jar with metal top used for gauze collection

**Figure 2:**
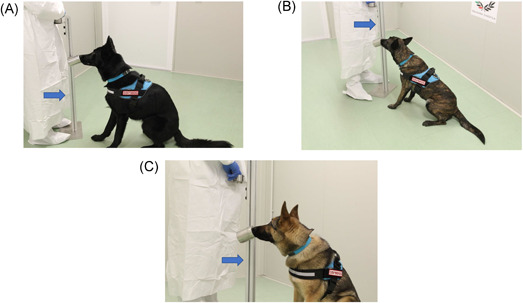
The sniffer stand (indicated by the arrow) used for the olfactory conditioning of the dog Harlock (A), Roma (B), and Idra (C)

**Figure 3:**
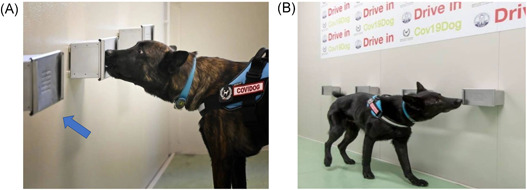
(A) Detection box (indicated by the arrow) in the panel. Different detection boxes used for the line‐up (B) set‐up during the olfactory discrimination research phase of the training protocol

The gauze was the elective support chosen for sweat collection, for its common distribution and consequent easy availability. The gauze used belongs to Class IIa surgical device for its specific characteristics to be sterile, 100% cotton, latex, and phtalate free (Figure 1A). Gauze with sweat was collected in a glass jar with the metal top (Figure 1B). For each collection, a new jar was used. The sniffer stand (Figure 2A–C) used for olfactory conditioning of dogs and the detection boxes (Fugure 3A) used for the line‐up (Figure 3B) were made of inert materials to avoid plastics or adhesive materials that could be confounding for the dog's sniff. In each phase of dog training, cross‐contamination was avoided from sweat collection, storage, transport to the training procedure. Training tools were projected to guarantee that the sweat samples never come in direct contact with dogs and the tools were carefully sanitized at the end of each training session.

### 2.4 Sweat samples collection procedure

Skin sweat samples from patients with COVID‐19 were collected at the COVID Center of the University Hospital Campus Bio‐medico of Rome, while skin sweat samples from patients without COVID‐19 were collected at the Internal Medicine Department of the University Hospital Campus Bio‐medico of Rome. The demographic and clinical characteristics of patients enrolled in the study are reported in Table 2. Samples were kept anonymous and data registered on a database were accessible only to the Principal Investigator of the study. Sweat samples were collected by patients with the assistance of the healthcare staff (physician or nurse) instructing them about the procedure, after the informed consent. This consisted of self‐collection by inserting gauze in the underarms, one for each side, and kept for 5 min. After this, the patient puts both gauzes in the same glass can, closed by the metal top, and gives it to the healthcare staff who puts it in a double bag for biological samples collection that could be delivered outside the COVID center to the Laboratory Division. Glass can with sweat samples were delivered to the dog's training space adhibited within the Drive‐in campus test area of the University Hospital Campus Bio‐Medico of Rome within 2 h from collection. If the training was delayed, the samples were stored in a refrigerator, under a controlled temperature of 4°C for 24 h at the maximum.

**Table 2:** Demographic and clinical characteristics of patients enrolled in this study

	Positive COVID‐19 patients *n* = 20	Negative COVID‐19 patients *n* = 15
Mean age (years)	57.8	73.4
Sex *n* (%)		
Male	9 (45)	10 (67)
Female	11 (55)	5 (33)
Comorbidity *n* (%)	9 (45)	15 (100)
Diabetes	2 (22)	0
High blood pressure	6 (66)	11 (73)
Cardiovascular disease	0	11 (73)
Obesity	3 (33)	0
Cancer	3 (33)	6 (40)
Ictus	1 (11)	0
COPD	0	4 (26)
Healthy *n* (%)	11 (55)	0
Diagnosis at admission		
COVID‐19 pneumonia	20 (100)	0
Pneumonia no COVID‐19	0	6 (40)
Cardiovascular disease	0	3 (20)
Ascites	0	1 (6.6)
Diverticulitis	0	1 (6.6)
Pleural effusion	0	2 (13)
Sepsis	0	2 (13)

### 2.5 Dog training description

During training, all operations of samples management were performed by healthcare staff adequately equipped with personal protective equipment (PPE), including single‐use water‐repellent lab coats, FFP3 disposable mask, water‐repellent cover shoes, nitrile gloves, and face shield. The same PPE was also used by the dog trainers in any phase of the training. Dogs were equipped with working dog equipment, including dedicated collars and gears daily sanitized.

The training was performed indoors in a dedicated and well‐equipped space consisting of a container located within the Drive‐In Covid test area of the University Hospital Campus Bio‐medico of Rome. Local temperature was controlled and maintained within a temperature range of 20–25°C to minimize the influence on the experimental assay. The same temperature range was checked and requested also for sweat samples used for dog training.

Sweat samples collected in the glass jar after removal of the metal top were put inside the metal boxes used for line‐up set‐up and placed for 5 min before test start, time required for trace “aging.” After this interval time, the sweat sample could be sniffed by dogs and the test began. At the end of the test, lasting about 2 min and 30 s, boxes with the sweat sample were alternatively moved within the line‐up and sanitized for dog rotation.

**Figure 4:**
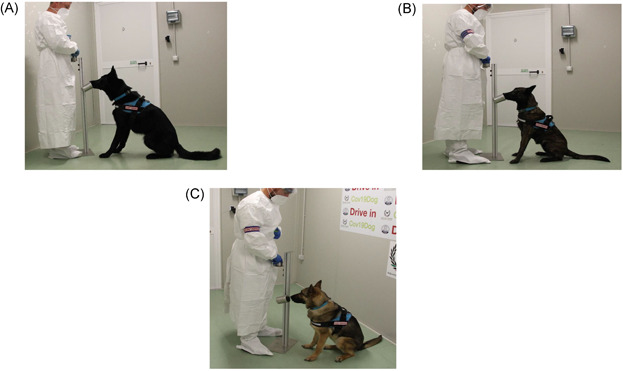
Ad hoc sniffer stand designed for the safe accommodation of the sweat sample and the comfortable positioning for the dog's trainer during the olfactory conditioning phase of the dog training protocol for Harlock (A), Roma (B), and Idra (C)

During the specific conditioning to COVID‐19 VOCs, an ad hoc designed sniffer stand (Figure 4A–C) has been used, allowing the safe accommodation of the sweat sample and the comfortable positioning for the dog's trainer when he enforced the correct behavior of the dog by the clicker and the material reward. In this phase, it is fundamental to use various sweat samples collected from different SARS‐CoV2 positive patients to fix in the olfactory memory of dogs the presence of VOCs specific for COVID‐19 within the vast range of odor emanations of the skin sweat. For the specific conditioning, sniff repeats have been performed with positive reinforcement by material rewards at each sniffing.

During the olfactory discrimination research the training aimed at the detection of COVID‐19 VOCs towards dog has been conditioned and the discrimination from what it is not of interest, that has to be extinguished. In this phase, detection boxes with sweat samples from SARS‐CoV2 positive patients, sweat samples from COVID 19 negative patients, blank gauze, and an empty box have been used. Sweat samples were hidden from the beginning and were never visible to the dogs, and were positioned randomly with the help of random software.

The basis of this training phase was in the handler‐dog K‐9 unit, where the handler realized that the dog was ready for the autonomous detection through the line‐up and for the correct and univocal response by “sitting” or “lying down.” This training phase was repeated 10–20 times to fix the correct behavior, depending on the dog's ability. This phase followed the verification of the training procedure by dedicated trial sessions for each dog during which the number of correct and incorrect identification of SARS‐CoV2 positive sweat samples was recorded for further statistical analysis of data.

### 2.6 Statistical analysis

The percentage difference between correct and incorrect identification registered in the verification of the protocol procedure for each dog was evaluated by *χ* ^2^ test for proportions. A *p* value of less than 0.05 was considered statistically significant.

### 2.7 De‐briefing session

Each training session has been video‐recorded (as in the supplementary material) for further analysis during the de‐briefing section performed at the end of each training session. Moreover, a specific report has been made for the training sessions tracing.

### 2.8 Sanitizing

Training site and training equipment were daily sanitized at the end of each training session, while wastes were disposed of in special waste containers.

### 2.9 Troubleshooting

Sweat self‐collection by inserting gauze in the underarms of the patients has to be carefully performed, as previously described, to avoid any influence on training.

## 3 RESULTS

**Figure 5:**
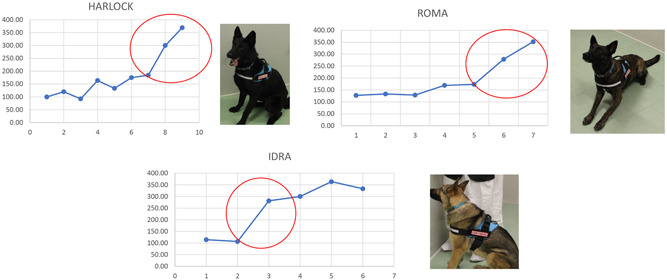
The gradual progression of the dogs until the switch moment (red circle): percentage of correct identification normalized for the coefficient of difficulty (Y‐axis) and the number of trials (X‐axis)

In April 2021 was completed the 4 weeks intensive training including 227 sessions and 700 tests with 92 different biological samples. These sessions aimed to identify the moment of switch for the dog. The switch is the time frame where the dog passes from a not relevant to a relevant percentage of correct reporting that has been fixed to 80% to be comparable to the gold standard diagnostic molecular and antigenic SARS CoV2 tests. From the switch, the training focused on fixing the correct behavior otherwise the most reliable reporting by the dog, as much as possible near to 100%. The gradual progression of the dogs in these sessions until the switch moment has been schematized is shown in Figure 5. Exactly, after assigning a coefficient of difficulty for each training session that is directly proportional to the number of boxes in the line‐up, it was observed that the dog Harlock from a minimum of 46% of correct reporting reached a maximum of 92%, the dog Roma from a minimum of 63% arrived at 92%, and the dog Idra from 53% pass to 100% of correct reporting. The number of specific trials performed from the switch moment for Harlock, Roma, and Idra is reported in Table 3. The occurred dog switch was evidenced in these trial sessions, exactly 17 for Harlock, 20 for Roma and 23 for Idra as reported in Table 3. Harlock correctly identied the SARS‐CoV2 positive sweat samples in the line‐up 15/17 (88%), Roma 17/20 (85%) and Idra 20/23 (87%) times. The difference between the percentage of correct and uncorrect identifications was statistically significant (*p* < 0.0001) (Table 3), confirming the switch obtained for each dog.

**Table 3:** Number (no.) and percentage (%) of total trials performed and correct and incorrect identification by the dogs for the verification of the training protocol

Dog's name	Trial no.	Correct *n* (%)	Uncorrect *n* (%)	*χ* ^2^	*p* Value
HARLOCK	17	15 (88)	2 (12)	19.06	<0.0001
ROMA	20	17 (85)	3 (15)	19.11	<0.0001
IDRA	23	20 (87)	3 (13)	24.64	<0.0001

## 4 DISCUSSION

Usually, the test used for a mass screening mass should be rapid, enough sensitive, easy to manage, cheap, and not time‐consuming. The use of olfactory dogs has been proposed as a fast, reliable, and not expensive tool. The most critical factor is to provide a training protocol for sniffer dogs that could be easy to perform and enough reliable. The protocol proposed in this study provided some causes for reflection about the understanding of the “switch” moment for the dog. This moment corresponds to the time where the dog passes from a not relevant to a relevant percentage of correct reporting, which is comparable to the gold standard diagnostic SARS CoV2 tests. In this study, data on the switch have been collected and the occurred dog switch evidenced in further trials sessions for each dog, with promising results in terms of sensitivity and specificity. Now the dog is ready for the mass screening in real‐life. Overall, the proposed protocol, with focus on the switch moment, could represent a valid support for sniffer dogs training replication in any setting.

The application of the proposed protocol for COVD‐19 dog alert by sniffing axillary sweat samples, confirmed the ability of dogs, after specific training to detect COVID‐19 VOCs. This approach provides a promising tool for COVID‐19 mass screening at airports, stadiums, or in case of crowded events where the virus transmission control by asymptomatic individuals is fundamental in public health. After this first step, future perspectives will include training of further dogs using odorless supports for skin emanation collection, the comparison between sniffer dogs ability and molecular RT‐PCR gold test for COVID‐19 diagnosis in different settings as the Drive‐In and the use of SARS‐CoV2 proteins for dogs training to direct viral particles instead of VOCs from sweat samples.

## CONFLICT OF INTERESTS

The authors declare that there are no conflict of interests.

## AUTHORS CONTRIBUTIONS

**Silvia Angeletti** and **Massimo Ciccozzi:** study design, data analysis, and study supervision. **Francesco Travaglino, Silvia Spoto, Maria Chiara Pascarella, Giorgia Mansi**, and **Raffaele Antonelli Incalzi:** patient clinical diagnosis and sweat samples collection. **Marina De Cesaris** and **Marta Fogolari:** COVID‐19 laboratory test. **Marta Giovanetti:** data analysis. **Silvia Sartea:** Logistic at the Drive‐in Area. **Davide Plescia** and **Massimiliano Macera:** study design and dog training. All authors contributed to the manuscript preparation.

## Supporting information

Supporting information.

Supporting information.

Supporting information.

Supporting information.

## Data Availability

Data are available in article Supporting Information Material.
