# Supplementary material for: COVID‐19 sniffer dog experimental training: Which protocol and which implications for reliable sidentification?
Source: J Med Virol. 2021 Jun 26;93(10):5924–30. doi: 10.1002/jmv.27147 (PMC8426906; doi:10.1002/jmv.27147)
Supplement: Supplementary file 4 — Supporting information. [file JMV-93-5924-s002.docx]

**Supplementary material**. Video-recording of the training sessions for the dog Harlock, Roma and Idra.
